# Cisplatin Effects on the Human Fetal Testis – Establishing the Sensitive Period for (Pre)Spermatogonial Loss and Relevance for Fertility Preservation in Pre-Pubertal Boys

**DOI:** 10.3389/fendo.2022.914443

**Published:** 2022-07-14

**Authors:** Gabriele Matilionyte, Michael P. Rimmer, Norah Spears, Richard A. Anderson, Rod T. Mitchell

**Affiliations:** ^1^ MRC Centre for Reproductive Health, The Queen’s Medical Research Institute, The University of Edinburgh, Edinburgh, United Kingdom; ^2^ Biomedical Sciences, The University of Edinburgh, Edinburgh, United Kingdom; ^3^ Department of Paediatric Diabetes and Endocrinology, Royal Hospital for Children & Young People, Edinburgh, United Kingdom

**Keywords:** human, fetal, testis, cisplatin, germ cell, fertility preservation

## Abstract

**Background:**

Exposure to chemotherapy during childhood can impair future fertility. Studies using *in vitro* culture have shown exposure to platinum-based alkylating-like chemotherapy reduces the germ cell number in the human fetal testicular tissues. We aimed to determine whether effects of exposure to cisplatin on the germ cell sub-populations are dependent on the gestational age of the fetus and what impact this might have on the utility of using human fetal testis cultures to model chemotherapy exposure in childhood testis.

**Methods:**

We utilised an *in vitro* culture system to culture pieces of human fetal testicular tissues (total n=23 fetuses) from three different gestational age groups (14-16 (early), 17-19 (mid) and 20-22 (late) gestational weeks; GW) of the second trimester. Tissues were exposed to cisplatin or vehicle control for 24 hours, analysing the tissues 72 and 240 hours post-exposure. Number of germ cells and their sub-populations, including gonocytes and (pre)spermatogonia, were quantified.

**Results:**

Total germ cell number and number of both germ cell sub-populations were unchanged at 72 hours post-exposure to cisplatin in the testicular tissues from fetuses of the early (14-16 GW) and late (20-22 GW) second trimester. In the testicular tissues from fetuses of mid (17-19 GW) second trimester, total germ cell and gonocyte number were significantly reduced, whilst (pre)spermatogonial number was unchanged. At 240 hours post-exposure, the total number of germ cells and that of both sub-populations was significantly reduced in the testicular tissues from fetuses of mid- and late-second trimester, whilst germ cells in early-second trimester tissues were unchanged at this time-point.

**Conclusions:**

*In vitro* culture of human fetal testicular tissues can be a useful model system to investigate the effects of chemotherapy-exposure on germ cell sub-populations during pre-puberty. Interpretation of the results of such studies in terms of relevance to later (infant and pre-pubertal) developmental stages should take into account the changes in germ cell composition and periods of germ cell sensitivity in the human fetal testis.

## Introduction

Chemotherapy treatment for cancer in boys is associated with impaired testicular function and impaired fertility (1). Human-relevant experimental models are important to determine the impacts of specific agents and the influence of developmental stage of the patient (2). We have utilised an *in vitro* system that has been previously shown to maintain testicular tissue integrity when exposing human fetal testicular tissue fragments in hanging drops, to clinically relevant concentrations of pharmaceutical compounds in order to determine the effect of exposure on somatic and germ cell populations (3–5). Using this system, we have previously shown that exposure to cisplatin, a platinum-based chemotherapeutic drug used in the treatment of several paediatric malignancies (6), results in a reduction in total germ cell number in cisplatin-exposed tissues (4).

Whilst spermatogenesis does not occur until adulthood, the germ cell number and composition within the testis undergo changes from fetal life through to puberty (7, 8). Therefore, effects of chemotherapy exposure prior to puberty will be dependent on the germ cell populations present and any changes in sensitivity to treatment. In the human fetal testis the germ cell complement consists of gonocytes and (pre)spermatogonia. Gonocytes predominate during the first trimester and differentiation from gonocyte to (pre)spermatogonia occurs during the second and third trimester (7, 9). During infancy the remaining gonocytes complete differentiation to spermatogonia and for the remainder of childhood, spermatogonia make up the germ cell complement of the testis (8). Therefore, effects of chemotherapy on gonocytes has relevance to the fetal and infantile testis, whilst effects on (pre)spermatogonia are relevant to the testis from late first trimester until adulthood.

A critical period of testicular development during fetal life, known as the masculinisation programming window (MPW), has been identified in rats, during which perturbation of androgens predisposes to the development of male reproductive disorders (10). This resembles the Testicular Dysgenesis Syndrome (TDS) described in human and a similar period of sensitivity to androgens has been proposed to occur between 8-13 weeks gestation (11) TDS includes disorders of germ cell development such as testicular cancer and infertility (12). Whether there are similar critical periods of germ cell sensitivity to chemotherapy in fetal life and if there are differential effects on the germ cell sub-populations during human fetal testis development, has not been reported.

We aimed to determine whether there are specific periods of sensitivity to germ cell loss in the human fetal testis. If this is the case, it might affect how results of *in utero* chemotherapy exposure studies are interpreted. In addition, since the (pre)spermatogonia are the germ cell population present in the pre-pubertal testis, we aimed to determine whether there was a particular gestational age, where the reduction in (pre)spermatogonial numbers was observed.

## Methods

### Study Approval

Human fetal tissues were collected after women gave informed consent to donate tissue for research following elective termination of pregnancy (TOP). The work was conducted following ethical approvals (Edinburgh: South East Scotland Research Ethics Committee (LREC08/S1101/1), Newcastle: NRES committee North East – Newcastle and North Tyneside 1 (08/H0906/21+5) and London: NRES Committee London – Fulham (18/10/0822)).

### Tissue Collection

Human fetal testicular tissues from 2^nd^ trimester fetuses (total n=23; 14-22 GW) were obtained from elective TOP. Tissues were obtained from Royal Infirmary of Edinburgh and Human Developmental Biology Resource (HDBR) facilities in Newcastle and London. Samples from TOP with known fetal abnormalities were excluded. Gestational age of the fetus was determined by a combination of ultrasound and direct measurement of foot length. The sex of the fetus was confirmed by presence of sex-determining region Y (SRY) gene based on melting curves in qPCR runs using a small piece of skin or limb from each fetus. The number of samples that came from each site of TOP were as follows: London – 17, Newcastle – 4 and Edinburgh – 2. All samples were placed in media (Liebowitz L-15 with glutamine, 10% fetal bovine serum, 1% penicillin/streptomycin and 1% non-essential amino acids) and transported to Edinburgh in insulated boxes containing cool-packs. All samples were received within 72 hours of collection. All tissue culture experiments were performed in the same laboratory in Edinburgh.

### *In Vitro* Culture System

Tissue pieces (~1 mm^3^) were cultured in hanging drops containing 30 μl droplets of pre-warmed culture media on the upturned lid of Petri dish as previously described (5). Culture dishes were incubated at 5% CO_2_ at 37°C. Culture medium consisted of: Minimum Essential Medium α (MEMα; Lonza), 1x MEM non-essential amino acids (Thermo Fisher Scientific), 2 mM sodium pyruvate (Thermo Fisher Scientific), 2 mM L-glutamine (Life Technologies), 1x Insulin-Transferrin-Selenium (ITS; Sigma-Aldrich), 1x penicillin/streptavidin (Thermo Fisher Scientific) and supplemented with 10% fetal bovine serum (FBS; Life Technologies). The culture media was replaced every 24 hours. For treatments, media was supplemented with cisplatin (0.5 μg/ml) or vehicle (water) for 24 hours beginning on day 3 for 24 hours with analysis at 72 hours or 240 hours after cisplatin-exposure. To generate sufficient samples at each gestational age, we included data from experiments previously reported (4), with additional samples at each gestational age group.

### Tissue Processing

Cultured tissue pieces were fixed in Bouin’s fluid (Clin-Tech) for 1 hour prior to transfer to 70% ethanol. Fixed samples were embedded in paraffin, sectioned (5µm thickness) and morphology was assessed using H&E according to a standard protocol. All samples included in the analysis showed grossly normal morphology (healthy architecture of tubules, minimal apoptosis and presence of germ cells) in pre-culture and/or vehicle controls. For each treatment a minimum of two sections from two replicate tissue pieces were analysed.

### Immunostaining and Cell Quantification

Double colourimetric immunohistochemistry was performed according to previously published protocols (4). Co-staining was performed for expression of AP2γ (gonocytes) and MAGE-A4 ((pre)spermatogonia). Positive controls consisted of pre-culture tissue and negative controls and involved omission of the primary antibody. Positively stained germ cells were manually counted and reported per seminiferous area (mm^2^). The assessor remained blinded to treatment group during analysis. Details of antibodies and dilutions are provided in **Table 1**.

**Table 1:** Summary of immunohistochemistry protocol.

Antibody (Cat no)	Method	Dilution (antigen retrieval)	Origin	Blocking agent	Detection
**AP2γ** (sc-12762)	IHC	1:20 (citrate buffer)	Mouse	Normal horse serum/ TBS/BSA	DAB
**MAGE-A4** (Gift from Giulio Spagnoli)	IHC	1:40 (citrate buffer)	Mouse	Normal horse serum/ TBS/BSA	Vector Blue

### Statistics

For each treatment regimen, tissue from each fetus was considered an individual experiment. Fragments from each fetus were cultured and randomly allocated to receive vehicle or cisplatin. No outliers were excluded. Statistical analysis was conducted using GraphPad Prism 8 software (GraphPad Software Inc., USA). Two-way analysis of variance (ANOVA) with multiple comparisons using Bonferroni’s *post hoc* test was performed, accounting for treatment group (vehicle or cisplatin) and individual sample (fetus) as two independent variables (13). Data are presented as mean ± standard error of mean (SEM) for all fragments from each fetus and each distinct colour-coded point or square represents an individual fetus. Statistical significance was defined as *p*<0.05.

## Results

### Exposure to Cisplatin- Induced Germ Cell Loss in Human Fetal Testis Tissues

**Figure 1:**
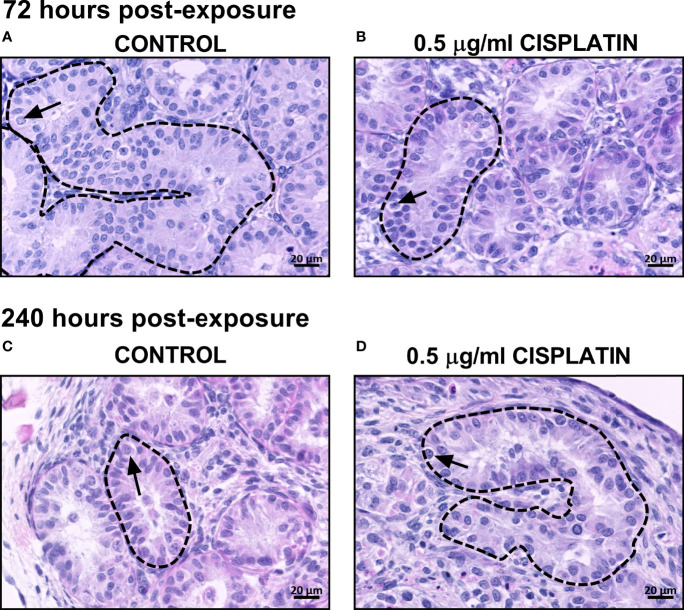
Effects of exposure to cisplatin on the gross morphology of human fetal testicular tissues at 72 and 240 hours post-exposure. H&E staining in vehicle control **(A, C)** and cisplatin-exposed **(B, D)** tissues at 72 and 240 hours post-exposure. Dotted lines outline seminiferous cords and arrows point to germ cells. Scale bars represent 20 μm.

**Figure 2:**
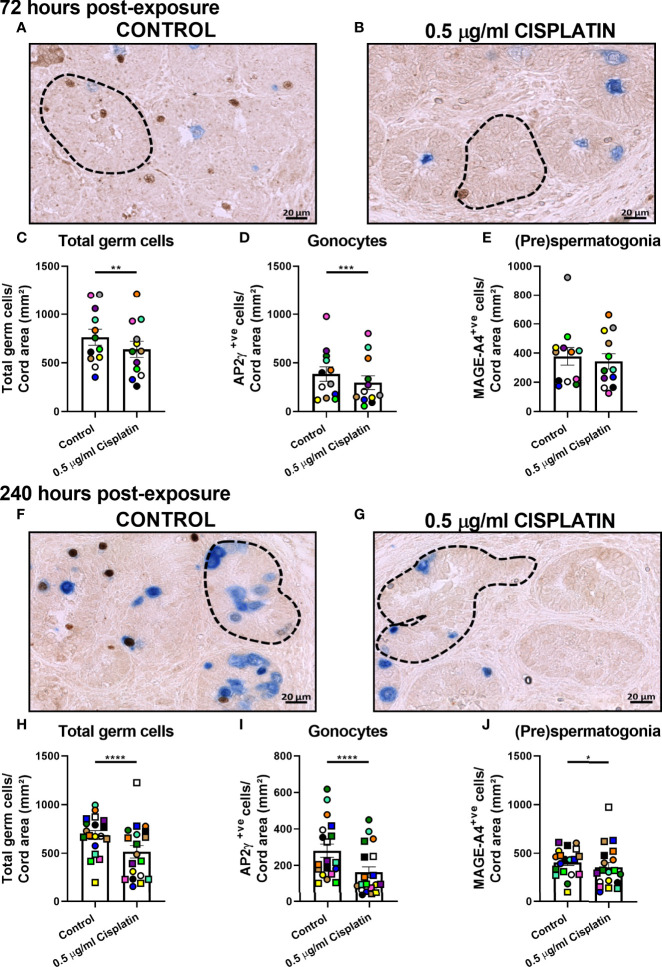
Pooled data on effects of exposure to cisplatin on germ cell number in human fetal testicular tissues at 72 and 240 hours post-exposure. Immunohistochemical staining for gonocytes (AP2γ+; brown) and (pre)spermatogonia (MAGEA4+; blue) in the human fetal testis in vehicle control **(A)** and cisplatin-exposed **(B)** tissues at 72 hours; and in vehicle control **(F)** and cisplatin-exposed **(G)** tissues at 240 hours post-exposure. Scale bars represent 20 μm. Dotted lines outline seminiferous cords. Total number of germ cells **(C)**, gonocytes **(D)** and (pre)spermatogonial **(E)** at 72 hours post-exposure. Total number of germ cells **(H)**, gonocytes **(I)** and (pre)spermatogonial **(J)** at 240 hours post-exposure. Data analysed using two-way ANOVA. *p < 0.05, **p < 0.01, ***p < 0.001, ****p < 0.0001. Values shown are means ± SEM and each data point represents the mean value for all fragments obtained from an individual fetus (n = 12-19).

Normal testicular morphology was maintained in control (**Figures 1A, C**) and cisplatin-exposed (**Figures 1B, D**) human fetal testicular tissues, with preservation of seminiferous cords and presence of germ cells at 72 (**Figures 1A, B**) and 240 (**Figures 1C, D**) hours post-exposure. Immunohistochemistry was carried out for AP2γ (gonoytes) and MAGE-A4 ((pre)spermatogonia) cells (**Figures 2A, B, F, G**) and used for quantification. At 72 hours, cisplatin exposure resulted in a significant reduction in the total number of germ cells (639 ± 82 vs 763 ± 83 cells/cord area (mm^2^), *p*<0.01; **Figure 2C**) and gonocytes (296 ± 70 vs 385 ± 74 cells/cord area (mm^2^), *p*<0.001; **Figure 2D**), whilst (pre)spermatogonial cell number (**Figure 2E**) was unchanged, compared to controls. At 240 hours, cisplatin exposure resulted in a significant reduction in total germ cell (516 ± 64 vs 688 ± 45 cells/cord area (mm^2^), *p*<0.0001; **Figure 2H**), gonocyte (163 ± 30 vs 281 ± 36 cells/cord area (mm^2^), *p*<0.0001; **Figure 2I**), and (pre)spermatogonial cell (357 ± 50 vs 408 ± 33 cells/cord area (mm^2^), *p*<0.05; **Figure 2J**) number, compared to controls.

### Cisplatin-Induced Germ Cell Loss is Dependent on the Gestational Age of the Fetus

**Figure 3:**
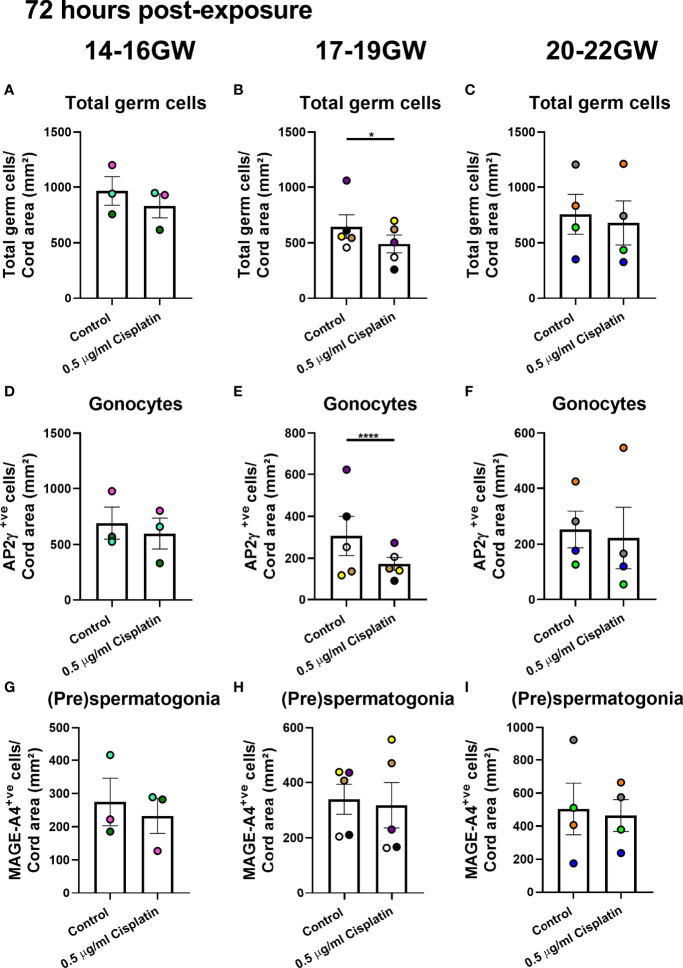
Effects of exposure to cisplatin on germ cell number in human fetal testicular tissues at 72 hours post-exposure according to gestational age. Total number of germ cells in early- **(A)**, mid- **(B)** and late- **(C)** second trimester human fetal testis. Gonocyte number in early- **(D)**, mid- **(E)** and late- **(F)** second trimester human fetal testis. (Pre)spermatogonial number in early- **(G)**, mid- **(H)** and late- **(I)** second trimester human fetal testis. Data analysed using two-way ANOVA. *p < 0.05, ****p < 0.0001. Values shown are means ± SEM and each data point represents the mean value for all fragments obtained from an individual fetus (n = 12).

The (pre)spermatogonial number is unchanged at 72 hours and only marginally reduced at 240 hours. Given the importance of this population when extrapolating results from the fetal testis to human pre-pubertal testis, we analysed according to three gestational age groups (early-, mid- and late-second trimester. At 72 hours post-exposure to cisplatin, there was no difference in total germ cell number or the germ cell sub-populations from fetuses obtained during early (14-16 GW; **Figures 3A, D, G**) or late (20-22 GW; **Figures 3C, F, I**) second trimester testicular tissue. However, there was a reduction in total germ cell number (491 ± 80 vs 646 ± 106 cells/cord area (mm^2^), *p*<0.05; **Figure 3B**) in mid-second trimester samples in the cisplatin-exposed tissues, compared to control. A differential effect on the sub-populations was demonstrated at this gestation with a significant reduction in gonocytes (173 ± 31 vs 307 ± 94 cells/cord area (mm^2^), *p*<0.0001; **Figure 3E**), whilst the number of (pre)spermatogonial (**Figure 3H**) was unchanged in cisplatin-exposed tissues, compared with control.

**Figure 4:**
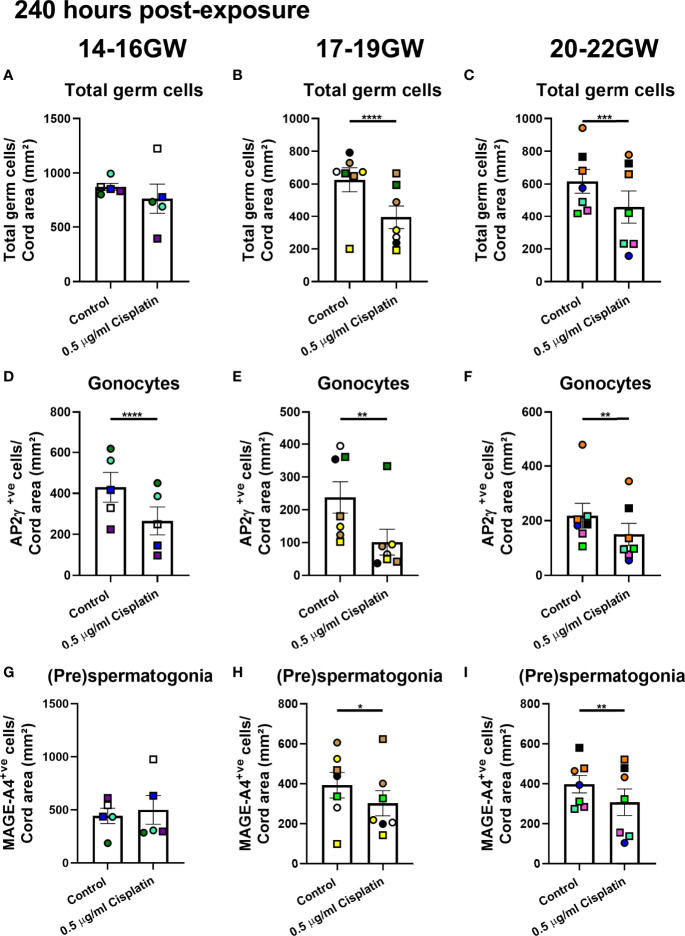
Effects of exposure to cisplatin on germ cell number in human fetal testicular tissues from different gestational age groups at 240 hours post-exposure according to gestational age. Total number of germ cells in early- **(A)**, mid- **(B)** and late- **(C)** second trimester human fetal testis. Gonocyte number in early- **(D)**, mid- **(E)** and late- **(F)** second trimester human fetal testis. (pre)spermatogonial number in early- **(G)**, mid- **(H)** and late- **(I)** second trimester human fetal testis. Data analysed using two-way ANOVA. *p < 0.05, **p < 0.01, ***p < 0.001, ****p < 0.0001. Values shown are means ± SEM and each data point represents the mean value for all fragments obtained from an individual fetus (n = 19).

At 240 hours post-exposure to cisplatin, there was no difference in total germ cell number from fetuses obtained during early (14-16 GW; **Figure 4A**) second trimester. There was a significant reduction in gonocytes (266 ± 68vs 430 ± 73 cells/cord area (mm^2^), *p*<0.0001; **Figure 4D**) after exposure to cisplatin at this stage, whilst the (pre)spermatogonia were unaffected (**Figure 4G**). For mid-second trimester tissues, there was a significant reduction in total germ cell number (395 ± 71 vs 627 ± 73 cells/cord area (mm^2^), *p*<0.0001; **Figure 4B**), gonocytes (101.2 ± 39.5 vs 238 ± 48 cells/cord area (mm^2^), *p*<0.01; **Figure 4E**) and (pre)spermatogonia (303 ± 63 vs 393± 64 cells/cord area (mm^2^), *p*<0.05; **Figure 4H**) in cisplatin-exposed tissues, compared with control (**Figure 4B**). Similarly, for late-second trimester tissues, cisplatin exposure resulted in a significant decrease in total germ cells (458 ± 98 vs 616 ± 73 cells/cord area (mm^2^), *p*<0.001, **Figure 4C**), gonocytes (151 ± 40 vs 218 ± 45 cells/cord area (mm^2^), *p*<0.01; **Figure 4F**) and (pre)spermatogonia (308 ± 66 vs 398 ± 44 cells/cord area (mm^2^), *p*<0.01; **Figure 4I**).

## Discussion

The aim of this study was to determine whether cisplatin effects on the germ cell sub-populations was dependent on the gestational age of fetus from which the testicular tissues were obtained. The results in this study indicate the effects of cisplatin exposure on germ cell number in second trimester human fetal testis are restricted to the middle and late time-points. This suggests a specific period of susceptibility to germ cell loss following cisplatin exposure. These data may have implications for understanding the effects of chemotherapy treatment in pregnant women on the fetus. In addition, these findings are important when interpreting the results from studies that include tissues from fetuses across the whole gestational period and, moreover, are essential for the design of future studies aimed at using human fetal testis cultures to model the effects of chemotherapy on spermatogonia in the pre-pubertal testis.

### Impacts of *In Utero* Exposure to Cisplatin on Human Fetal Testis – Windows of Sensitivity of Germ Cell Sub-Populations

Chemotherapy administration during pregnancy may result in spontaneous abortion and fetal death. The potential for fetal death is highest in the first trimester and reduces as pregnancy progresses (14). In a large (n=1170 women) cohort study of obstetric and neonatal outcomes in women diagnosed with cancer during pregnancy, 88% of the pregnancies resulted in a livebirth (15). A recent review reported livebirth in all 36 pregnant women treated with platinum-based chemotherapy (16). However, despite the relatively high survival of chemotherapy-exposed fetuses, there are no reports on the impacts of intrauterine cisplatin exposure on gonadal development and function. Our previous study involving xenografting of cisplatin-exposed human fetal testis demonstrated that germ cell loss persists over time and the degree of germ cell loss (~50% reduction in cisplatin-exposed compared with control), is similar at 3 months in xenografts, to that observed after short-term culture (4). The present results indicate that gonocytes are susceptible to loss following cisplatin exposure during the majority of the second trimester. The persistent sensitivity of this cell population may impact on the ability of gonocytes to contribute to the (pre)spermatogonial pool. The present study also suggests that the most sensitive period for the (pre)spermatogonial population appears to occur during the middle to late stages of the second trimester. The difference in sensitivity between the germ cell populations may relate to variation in the proliferation rate, 20-30% in gonocytes compared with 0-5% in (pre)spermatogonia, between these two populations during the second trimester (17).

### Impacts of Chemotherapy Exposure of Human Fetal Testis as a Proxy for the Childhood Testis

Germ cell differentiation from gonocyte to spermatogonia occurs asynchronously during fetal and early postnatal life (7, 18). During the first trimester, the majority of germ cells are defined as gonocytes based on the expression of markers including POU5F1 (pluripotency factor) and AP2γ. During the second trimester however, the proportion of gonocytes reduces as the (pre)spermatogonial (MAGE-A4) population increases (7). In infancy, the remaining gonocytes differentiate into spermatogonia and subsequently the germ cell complement consists of spermatogonia, until the onset of spermatogenesis at puberty (8, 18). Therefore, extrapolation of the results of studies involving the *in vitro* human fetal testis model to the infant or childhood testis must take account of the germ cell composition and periods of sensitivity for the germ cell sub-populations. The present results demonstrate the importance of gestational age in designing studies to determine effects of chemotherapy-exposure or protection from chemotherapy-induced germ cell loss. Studies aimed at understanding the effects of chemotherapy exposure on the human infant and pre-pubertal testis, should focus on a relevant developmental age with respect to the (pre)spermatogonial population. The present results suggest that the optimal time-point for these human fetal testis tissues is from the mid-second trimester onwards.

## Conclusions

*In vitro* culture of human fetal testis can be a useful model system to investigate the effects of chemotherapy-exposure on germ cell sub-populations during pre-puberty. Interpretation of the results of such studies, in terms of relevance to later (infant and pre-pubertal) developmental stages, should take into account the changes in germ cell composition and periods of germ cell sensitivity in the human fetal testis.

## Data Availability Statement

The raw data supporting the conclusions of this article will be made available by the authors, without undue reservation.

## Ethics Statement

The studies involving human participants were reviewed and approved by Edinburgh: South East Scotland Research Ethics Committee (LREC08/S1101/1), Newcastle: NRES committee North East – Newcastle and North Tyneside 1 (08/H0906/21+5) and London: NRES Committee London – Fulham (18/10/0822). The patients/participants provided their written informed consentto participate in this study.

## Author Contributions

Conceived and designed the experiments: GM, RM. Performed the experiments: GM. Analysed the data: GM, RTM. Feedback on the manuscript: MR, NS, RA. Wrote the paper: GM, RTM. All authors approved the final submission. RA and RM jointly supervised PhD student GM.

## Funding

RTM is supported by a UK Research and Innovation (UKRI) Future Leaders Fellowship (MR/S017151/1). Experimental work was supported by UKRI (MR/S017151/1), Wellcome (Grant No. 098522) and Children with Cancer UK (15-198). This work was undertaken in the MRC Centre for Reproductive Health funded by the Grant MR/N022556/1.

## Conflict of Interest

The authors declare that the research was conducted in the absence of any commercial or financial relationships that could be construed as a potential conflict of interest.

## Publisher’s Note

All claims expressed in this article are solely those of the authors and do not necessarily represent those of their affiliated organizations, or those of the publisher, the editors and the reviewers. Any product that may be evaluated in this article, or claim that may be made by its manufacturer, is not guaranteed or endorsed by the publisher.
